# A rare case of difficult airway management in a Klippel-Feil syndrome pediatric patient with osseous torticollis undergone orthopedic surgery: Difficult airway in pediatric patient with torticollis

**DOI:** 10.1186/s12871-021-01341-6

**Published:** 2021-04-19

**Authors:** Xiaoqing Zhang, Jun Wang, Yajie Liu, Zhengqian Li, Bin Han

**Affiliations:** Department of Anesthesiology, Peking University Third Hospital, 49 North Garden Rd., Haidian District, Beijing, China

**Keywords:** Difficult extubation, Osseous torticollis, Airway edema, Tonsil hypertrophy, Occipito-cervical fixation, Pediatric, Syndrome

## Abstract

**Background:**

Orthopedic surgery for cervical torticollis poses potential threat to airway management both in tracheal intubation and extubation. Klippel-Feil syndrome (KFS) is a complex syndrome of osseous and visceral anomalies. The anatomical characteristics of KFS might have significant implications for airway management.

**Case presentation:**

This is a rare case of an 8-year-old boy presenting with osseous torticollis, congenital occipito-atlantal deformity, congenital basilar invagination and KFS undergone elective torticollis correction surgery. Though with difficulty, tracheal intubation was successfully performed. Extubation failed twice on postoperative day 2 and 10, and required tracheostomy. Based on radiological findings, we speculated that prolonged airway edema accounted for the main reason of the failed extubation, the hypertrophic tonsil and occipito-cervical fusion resulted in reduced oropharyngeal space and limited cervical range of motion. Moreover, the Chiari malformation and KFS complicated the airway condition and lead to prolonged airway obstruction. The tracheostomy casing was removed 1 month later.

**Conclusions:**

Cautions should be taken in extubation of pediatric patients undergone major osseous torticollis surgery. Reintubation should be prepared in case of failed extubation. Severe post-operative airway edema, complicated with hypertrophic tonsil, the structural abnormalities in the oropharyngeal cavity, and occipito-cervical deformities constituted the decreased oropharyngeal space and resulted in failed extubation. For severe airway compromise and prolonged intubation, tracheostomy should be considered.

## Background

Cervical torticollis is a rare deformity characterized by a lateral head tilt and chin rotation toward the side opposite to the tilt. Klippel-Feil syndrome (KFS) is a cervical abnormalities in pediatric patients caused by the absence or fusion of cervical vertebrae. KFS was first reported in 1912 as a triad of short neck, restricted motion of the neck, and low posterior hairline. As a consequence of failure in normal segmentation of cervical vertebrae during the early weeks of fetal development, two or more of the seven vertebrae are fused. It has a rare incidence of 1:42000 births [1, 2]. The atlanto-occipital joint and spinal canal stenosis could co-exist in KF syndrome [3]. The surgical treatment of the cervical osseous deformity poses potential threat to airway management both in tracheal intubation and extubation. We reported a unique case of a pediatric patient undergone orthopedic surgery who need tracheostomy as a result of failed tracheal extubation. We will discuss the airway management of this patient and suggest possible causes in order to provide strategies for future events. Written informed consent was obtained from the guardian of the patient.

## Case presentation

An 8-year-old boy (weighing 34 kg) was found to be torticollis at age 1. He was diagnosed with osseous torticollis in our hospital, and was scheduled for elective orthopedic surgery.

### History

The boy has tonsil hypertrophy and sleep dyspnea in supine position and was scheduled for tonsillectomy in local hospital 1 year ago. The surgery was cancelled because of failed tracheal intubation. No special birth history was documented. No other dysplasia were reported.

### Pre-operative examination

#### ECG

Sinus arrhythmia by wandering pacemaker with sinoatrial node; incomplete right bundle branch block.

Carotid artery and vertebral artery ultrasound: no left common carotid artery was found (suspected with congenital variation).

#### Cervical CT and MRI examinations

Atlanto-occipital fusion; congenital skull base depression; atlantoaxial joint dislocation; cervical spine developmental malformations (C2-3 block vertebrae; C6 left appendage hypoplasia; C6-7 invisible cleft); sub tonsillar hernia.

#### Airway evaluation

**Fig. 1:**
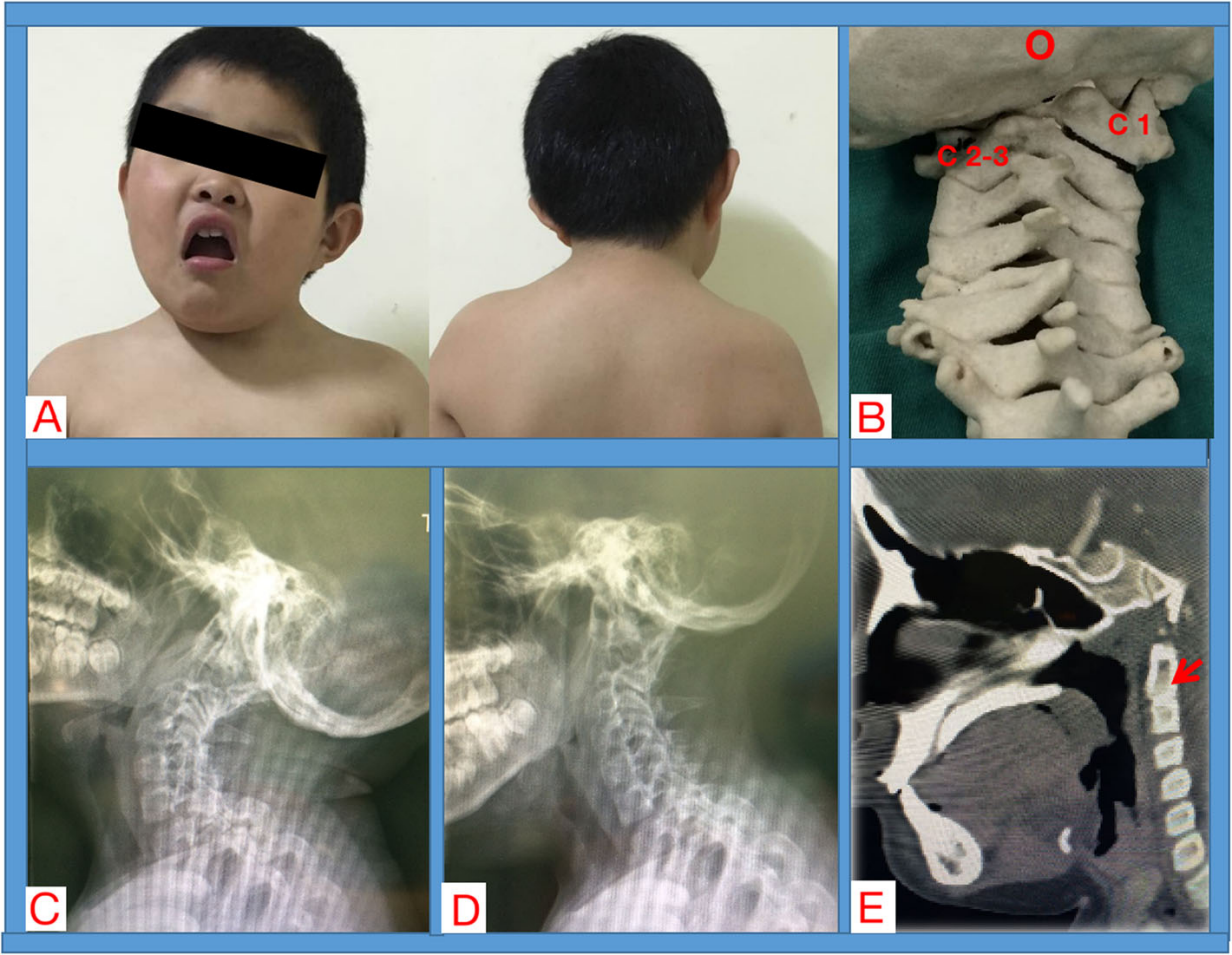
**a**, preoperative appearance of the pediatric patients with osseous torticollis. **b**, a Three-dimensional printed cervical vertebrae model of the pediatric patients with osseous torticollis. **c**, preoperative X-ray imaging of the lateral view of the cervical in extension position. **d**, the lateral view of the cervical X-ray imaging in reflection position. **e**, CT imaging showing the cervical spine developmental malformations (red arrow: C2-3 block vertebrae) (C, cervical vertebrae)

Modified Mallampati test was III ~ IV. The mouth opening degree (less than 3 fingers) and head and neck movement (60-120 degree) were limited. No significant narrowing of tracheal lumen and airway tortuosity were noticed in preoperative radiological findings (Fig. 1). The Colorado Pediatric Airway Score (COPUR score) with prediction points was 11 (C, points 1; O, points 2: with limited mouth opening, 20-40 mm; P, points 4: past failed intubation history; U, points 2: partially visible uvula; R, points 2: limited range of cervical motion, 60-120 degree). The glottic view prediction was 3, and the COPUR prediction points of 11 was indicative of “difficult intubation, fiberoptic less traumatic” [3]. No mandible/maxilla deformity or other hypoplasia were noted. Therefore, though with expected intubation difficulty, intubation was attempted under full preparation with difficult airway management tools. An experienced anesthesiologist with two assistant anesthesiologists were in charge of this case.

The patient’s periphery oxygen saturation (SpO_2_) was 100% breathing room air, in supine position. Dexamethasone was given to prevent postoperative nausea and vomiting, as well as airway edema. After sedated with propofol (with fractioned dose of 80 mg) following preoxygenation, sufficient facemask ventilation was confirmed. The spontaneous breathing was retained to ensure the absent of difficult intubation condition. The patient’s epiglottis was revealed during sedation. However, after administering 10 mg atracurium and 10 μg sufentanyl, the facemask ventilation suddenly became impossible. A reinforced endotracheal tube (I.D. of 6.0 mm) was tried under glidescope but failed. The SpO_2_ dropped to 60. Direct laryngoscopy with medium sized Macintosh blade was tried again by another attending anesthesiologist after optimizing ventilation maneuvers. A reinforced endotracheal tube with sealing cuff (I.D. of 5.5 mm) was placed successfully. The whole intubation duration was approximately 12 min.

After prone position was made by orthopedic surgeons with continuous cervical traction (Mayfield®, Integra LifeScience. Inc., US), orthopedic surgery was performed, including the exploration anteriorly of the wedged C2 and C3-4 intervertebral disk, the fixation posteriorly of occipital-C4 pedicle with iliac bone graft, and correction of cervical torticollis. The surgical duration was 334 min and the hemodynamic status was stable during the whole procedure. A total of 1700 ml crystalloid solution was infused, with intraoperative blood loss of 200 ml, and autologous blood transfusion of 100 ml washed red blood cells. The boy was transferred to the ICU with continuous sedation and analgesia without extubation.

Extubation was normally performed on post-operative day 1 in fear of prolonged intubation related complications such as ventilator associated pneumonia or airway complication. After positive result of cuff-leak test, ICU doctors postponed the extubation and invited anesthesiologists for consultation the next day. On postoperative day 2, anesthesiologist and ICU doctors performed cuff leak test. The result was still positive. Considering the success of the first intubation, extubation was attempted with preparation of re-intubation. The patient suffocated immediately after extubation despite complete consciousness and with normal blood-gas analysis results. Inspiratory stridor and cyanosis was noted soon after. Dexamethasone was administered to reduce airway edema and other possible intubation related damage. The patient’s heart rate (HR) dropped from 125 bmp to 80 bpm during re-intubation. Epiglottis and laryngeal edema were revealed by video laryngoscope, but the glottis could not be visualized. Tracheal re-intubation finally succeeded 17 min later under propofol sedation.

**Fig. 2:**
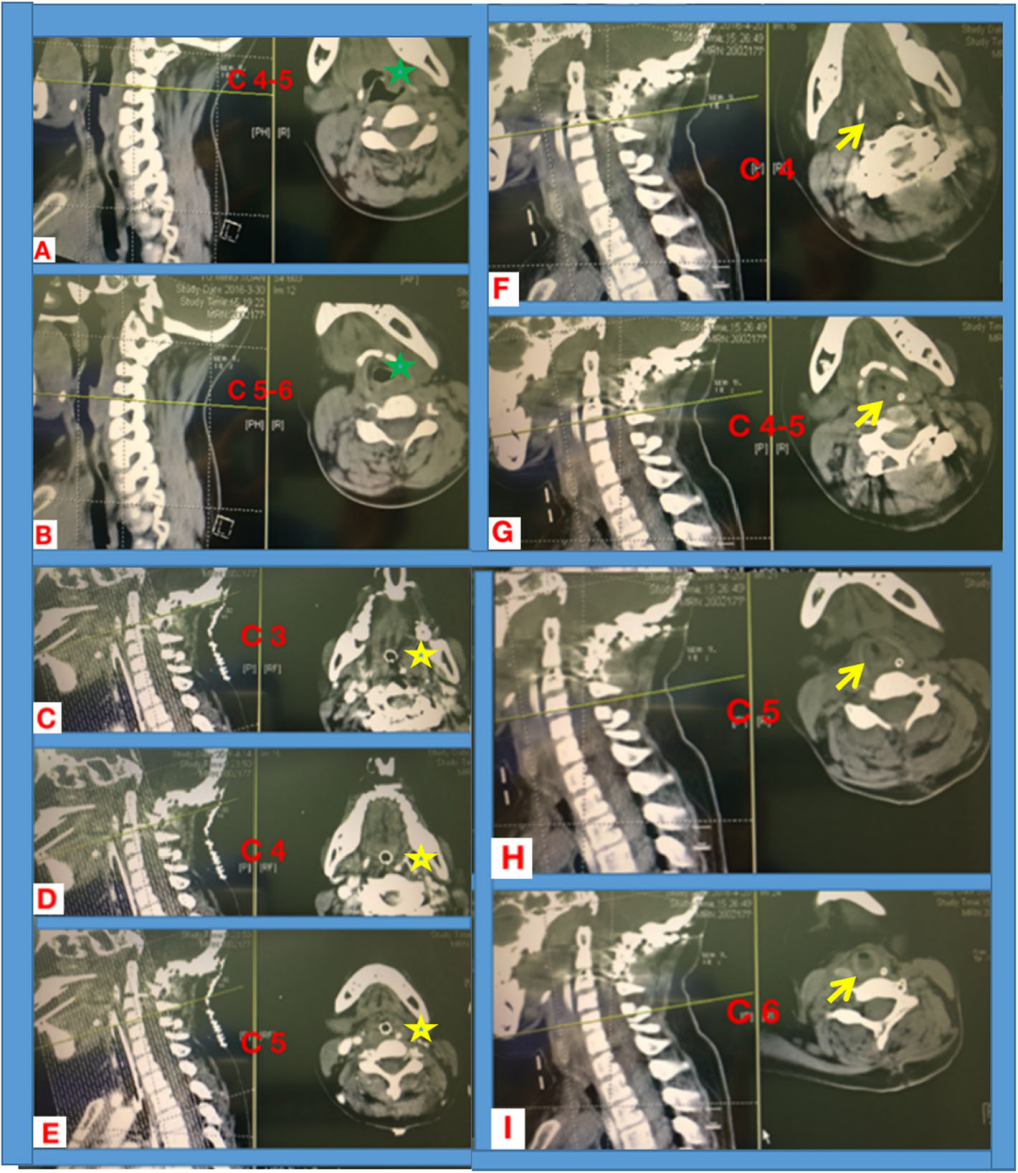
Cervical coronal and corresponding transverse section imaging of CT scanning. Preopeartive imaging showing no obstruction in upper airway in cervical vertebrae 4-6 segement (**a**, Cervical vertebrae 4-5 and **b**, Cervical vertebrae 5-6; green asterisk, trachea). On postoperative day 9, no space between tracheal tube and surrounding tissue was noticed from CT scanning images, indicating severe airway edema (**c**-**e**, corresponding to Cervical vertebrae segement of 3-5; yellow asterisk, showing swollen Oropharyngeal cavity). On postoperative day 15 (5 days after trecheostomy), airway stenosis still existed with significant edema, corresponding to the level of Cervical vertebrae segement of 4-6, (**f**-**i**; corresponding to C4-6 segement; yellow arrow, airway edema and resultant tracheal stenosis)

CT scanning was made on postoperative day 9 which revealed airway edema comparing with preoperative findings (Fig. 2c-e). The extended intubation duration and long-term sedation would lead to ventilator associated pneumonia and complicated airway management. After Fiber-optical bronchoscope (FOB) inspection showing alleviated airway edema on postoperative day 9, and the potential existence of deep cervical fascial space from CT imaging, a proposal of attempted extubation in the operating room and tracheotomy as the alternative plan was made by multidisciplinary consultation including orthopedics, anesthesiologists, otolaryngologists, and ICU physicians. On postoperative day 10 in the operating room, the patient was extubated after fully suction of the sputum and sufficient oxygenation. However, inspiratory dyspnea soon ensued. The patient was put in left lateral head-up position with facemask oxygenation and placement of oropharyngeal airway device. Dyspnea was not alleviated and cyanosis appeared 5 min after extubation. SpO_2_ dropped to 60% with HR of 70 bmp. Considering the existence of upper airway obstruction, re-intubation was performed immediately using glidescopy under propofol sedation. Tracheotomy was successfully performed under general anaesthesia with orthopedists’ assistance with better positioning the head and neck. The patient returned to ICU with vital signs stable and was weaning from mechanical ventilator the next day. Five days after tracheotomy, CT examination showed the still existence of the upper airway edema (Fig. 2f-i). Ten days later, the patient was transferred to the orthopedic ward.

One month after the first operation, the tracheostomy tube was blocked and no discomfort was complaint. Four days later, the tracheostomy casing was removed. The boy was discharged from hospital 3 days later. Three year follow-up revealed satisfactory orthopedic results.

## Discussion and conclusion

The boy was diagnosed with osseous torticollis, congenital occipito-atlantal fusion, congenital basilar invagination, atlanto-axial joint dislocation, KFS at C2-3, vertebral aplasia (C6), spina bifida occulta (C6-7), and Chiari Malformation (CM) in our hospital. It is worth to note that the extubation failed twice and finally required a tracheostomy. The possible reasons for difficult airway management were discussed in the following.

KFS is a complex syndrome of osseous and visceral anomalies. The anatomical characteristics of KFS have significant implications for intubation. It is characterized by failure in segmentation of two or more cervical vertebrae, with 75% congenital fusion cases occur in the first three cervical vertebrae [4]. Sporadic case reports showed the difficulties in repeated intubation attempts and associated complications and even abandonment of the surgical procedure. The cervical fusion present in KFS worsens with age as the spine reaches full maturity, and that clinicians should use available imaging modalities, anesthetic history, and comprehensive, preoperative airway examination to determine the potential risk of difficult airway [5]. In the present case, though with difficulty, the tracheal intubations were all successfully performed. However, the attempted extubations were failed and required emergent re-intubation and finally tracheostomy.

The rarely occurred airway obstruction after cervical spine surgery has been reported with swelling and hematoma. Laryngeal edema presents as inspiratory stridor within 30–60 min of extubation, and it is an important cause of post-extubation obstruction [6]. Normally, the degree of swelling peaked on day 2-3 after surgery, and then showed a gradually decreasing tendency [7]. Experience from our tertiary center is that the extubation could be safely performed immediately after cervical surgery. While for some major orthopedic surgery, it might take 24-48 h for safe extubation. In the current case, the delayed airway obstruction still existed 2 weeks after surgery and required tracheostomy. The upper airway edema is obvious from postoperative CT imaging and gradually dissipated after 1 month. For pediatric patients, large adenoids/tonsils and obese are important indications for difficult airway management [8]. Considering the patient’s past medical history of sleep dyspnea in supine position due to tonsil hypertrophy, the failed tracheal intubation and the canceled tonsillectomy, we speculated that this might constitute as an important factor for prolonged extubation when complicated with airway edema.

Moreover, the occipito-cervical alignment has significant impact on the oropharyngeal space. Sporadic case reports revealed that the angle of occipito-cervical (O-C) fusion would be critical for postoperative upper airway obstruction [9]. A retrospective clinical study showed there’s a difference in the O-C2 angle (the angles between the inferior endplate of C2 vertebrae body and the McGregor’s line). The percentage changes in the cross-sectional area of the oropharynx before and after surgery were linearly correlated. Deceased O-C2 angel may lead to dyspnea and/or dysphagia after surgery [10]. For patients with occipital-cervical fixation surgery, extubation should be performed with caution.

**Fig. 3:**
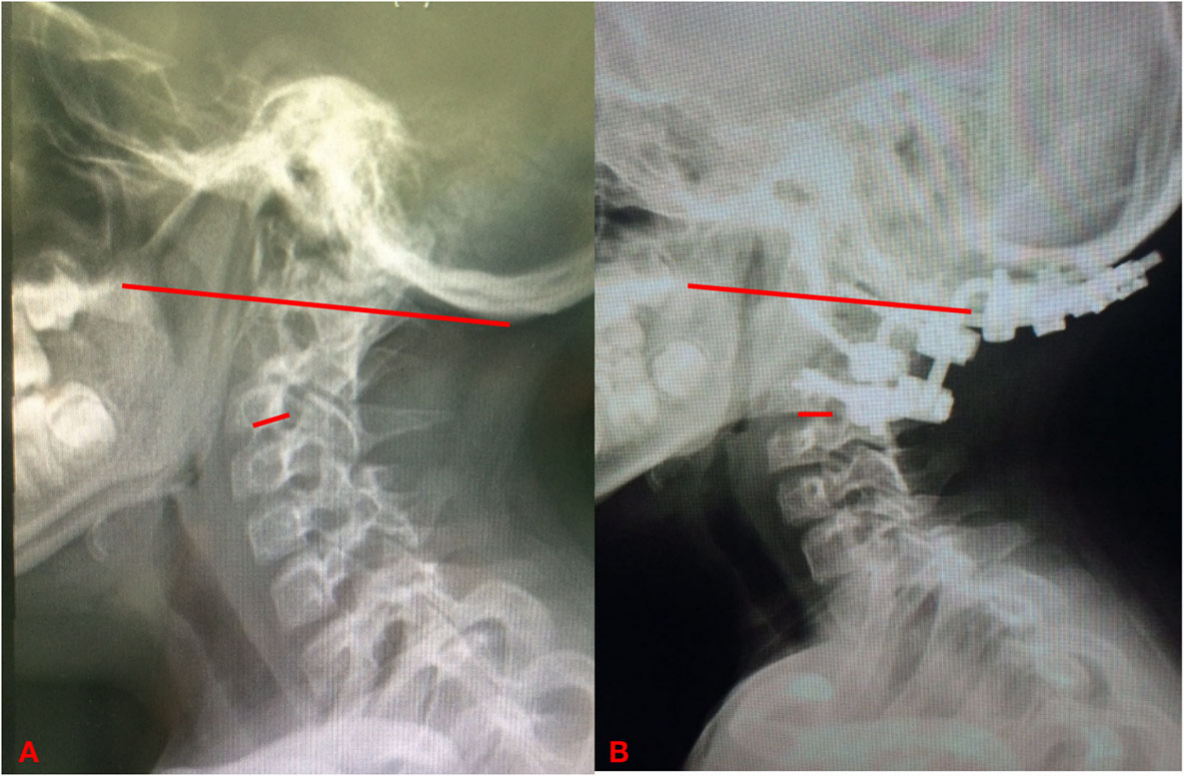
It is hard to make an accurate measurement of O-C4 angle as this patient had complex cervical abnormalities (congenital occipitoatlantal fusion, congenital basilar invagination, atlanto-axial joint dislocation, KFS at C2-3, and the subsequent fixation of O-C4). We made a rough estimation of the O-C4 angle (the angel between the long and short red lines) from lateral X radiography of preoperative (**a**) and postoperative (**b**) (2 months after surgery) in neutral position. A slight reflection of the O-C4 was noticed when compared with preoperative imaging (O, occiput; C, cervical vertebrae)

The boy’s congenital cervical skeletal deformity including the basal invagination and KFS, as well as the internal fixation may interfere with the accurate measuring of O-C2 angel. Therefore, we could only made a rough estimation of the O-C4 angel. However, from lateral view of the cervical X-ray in neutral position, a slight decrease of the O-C4 angel was noticed (Fig. 3). The O-C4 fusion immobilized the head and neck movement, and forced the patient’s head in a slight flexion position when compared with preoperative imaging, which further compressed the laryngeal space and resulted in the failed extubation. Moreover, pediatric patients with large tonsils, adenoids or obesity were prone to airway collapse during anaesthesia [11]. The decreased oropharyngeal space caused by the surgery were further compressed by the postoperative laryngeal edema and tonsil hypertrophy which ultimately resulted in difficulty breathing and tracheostomy.

Chiari Malformation (CM) is the downward herniation of the caudal part of the cerebellum and/or medulla oblongata into the spinal canal, and is associated with basilar invagination and can alter neurological functions such as upper airway motility and respiratory control, not only central, but also obstructive respiratory events [12]. In this case, the tonsil hypertrophy and CM may not directly lead to airway obstruction, but may complicate the difficulty airway management. The compensatory range of the upper airway space in this patient was limited with the pre-existing tonsil hypertrophy as well as changed upper airway muscle tension caused by the Chiari deformity. The post-operative decreased O-C4 angel further worsened the condition and might be an important constitutional factor in postoperative dyspnea [13].

Extubation should be performed with caution. However, FOB examination and cuff leak test could only reflect subglottic airway condition. It is reported from a multicenter evaluation study that several cuff leak tests display limited diagnostic performance for the detection of post-extubation stridor. Given the high rate of false positives, routine cuff leak test may expose to undue prolonged mechanical ventilation [14–17]. Inappropriate selection of tracheal tube would lead to confused result. Therefore, we did not solely depend on this test. For the narrowing of the oropharyngeal space, more thorough examination including CT scanning should be performed. Multi-disciplinary consultation is important for deciding appropriate extubation time. Anaesthesiologists should get well prepared for the emergent airway condition. However, there is no pediatric-specific universal extubation guidelines or experts consensus. Current algorithms are modifications of adult approaches which are often inappropriate.

We reported a rare case of a pediatric patients with cervical osseous deformity undergone orthopedic surgery and need tracheostomy as a result of failed tracheal extubation. The prolonged upper airway obstruction after occipito-cervical fusion have never been reported in pediatric patients undergone osseous torticollis surgery. The causes of prolonged airway obstruction after occipito-cervical fusion are multifactorial. The upper airway edema constituted the major reason, and the hypertrophic tonsil and the congenital cervical malformation may further complicated the airway condition with limited occipital-cervical range of motion and decreased compensatory space of the oropharyngeal cavity.

Cautions should be taken during extubation process in pediatric patients who undergone major osseous torticollis surgery. Evaluation of general clinical factors that may produce an adverse impact on ventilation after tracheal extubation should be comprehensively considered and optimized. An extubation and possible re-intubation plan in case of failed extubation that can be implemented to guarantee adequate ventilation should be formulated in advance. We recommend bougie or exchange catheters to avoid “cannot intubate, cannot ventilate” condition. For severe airway compromise and prolonged intubation, tracheostomy should be considered.

The appearance of the patient was significantly improved at the 3 years follow up after discharge from hospital.

## Data Availability

All data generated or analyzed during this study are included in this published article.

## Abbreviations

KFS: Klippel-Feil Syndrome
O-C: Occipito-cervical
FOB: Fiber-optical bronchoscope
CM: Chiari Malformation
